# EGR1 suppresses HCC growth and aerobic glycolysis by transcriptionally downregulating PFKL

**DOI:** 10.1186/s13046-024-02957-5

**Published:** 2024-01-29

**Authors:** Mingang Pan, Muyu Luo, Lele Liu, Yunmeng Chen, Ziyi Cheng, Kai Wang, Luyi Huang, Ni Tang, Jianguo Qiu, Ailong Huang, Jie Xia

**Affiliations:** 1 Key Laboratory of Molecular Biology for Infectious Diseases (Ministry of Education), Chongqing Medical University, Chongqing, 400016 China; 2 Department of Hepatobiliary Surgery, The First Affiliated Hospital, Chongqing Medical University, Chongqing, 400016 China

**Keywords:** HCC, EGR1, PFKL, Glycolysis, Sorafenib

## Abstract

**Background:**

Hepatocellular Carcinoma (HCC) is a matter of great global public health importance; however, its current therapeutic effectiveness is deemed inadequate, and the range of therapeutic targets is limited. The aim of this study was to identify early growth response 1 (EGR1) as a transcription factor target in HCC and to explore its role and assess the potential of gene therapy utilizing EGR1 for the management of HCC.

**Methods:**

In this study, both in vitro and in vivo assays were employed to examine the impact of EGR1 on the growth of HCC. The mouse HCC model and human organoid assay were utilized to assess the potential of EGR1 as a gene therapy for HCC. Additionally, the molecular mechanism underlying the regulation of gene expression and the suppression of HCC growth by EGR1 was investigated.

**Results:**

The results of our investigation revealed a notable decrease in the expression of EGR1 in HCC. The decrease in EGR1 expression promoted the multiplication of HCC cells and the growth of xenografted tumors. On the other hand, the excessive expression of EGR1 hindered the proliferation of HCC cells and repressed the development of xenografted tumors. Furthermore, the efficacy of EGR1 gene therapy was validated using in vivo mouse HCC models and in vitro human hepatoma organoid models, thereby providing additional substantiation for the anti-cancer role of EGR1 in HCC. The mechanistic analysis demonstrated that EGR1 interacted with the promoter region of phosphofructokinase-1, liver type (PFKL), leading to the repression of PFKL gene expression and consequent inhibition of PFKL-mediated aerobic glycolysis. Moreover, the sensitivity of HCC cells and xenografted tumors to sorafenib was found to be increased by EGR1.

**Conclusion:**

Our findings suggest that EGR1 possesses therapeutic potential as a tumor suppressor gene in HCC, and that EGR1 gene therapy may offer benefits for HCC patients.

**Supplementary Information:**

The online version contains supplementary material available at 10.1186/s13046-024-02957-5.

## Background

Hepatocellular carcinoma (HCC), the third most common cause of cancer-related deaths worldwide and accountable for around 830,000 fatalities each year [1], represents a significant public health concern. The overall survival rate for HCC is notably low, with a median survival typically ranging from six to ten months [2, 3]. However, the treatment choices for advanced HCC are restricted [4]. Sorafenib stands as the sole FDA-approved initial systemic therapeutic medication for 10 years. Nevertheless, the effectiveness of sorafenib as a treatment for HCC is unsatisfactory [4]. The SHARP (Sorafenib Hepatocellular Carcinoma Assessment Randomized Protocol; NCT00105443) trial demonstrated that sorafenib increases the overall survival of patients with HCC by a mere duration of 2.8 months [5]. Therefore, it is crucial to quickly discover new therapeutic targets for HCC and tackle the problem of sorafenib resistance.

Transcriptional dysregulation serves as a distinguishing feature in numerous cancers, leading to abnormal gene expression. Within the realm of cancer, these abnormal gene expression profiles significantly contribute to the progression of various cancer states [6, 7]. Transcription factors, a group of DNA-binding proteins, act as the primary regulators of transcriptional programs, initiating the transcription of specific genes based on the cellular context. The functionality of transcription factors in cancer cells can either be oncogenic or tumor suppressive. Thus, the dysregulated transcription factors can potentially instigate the genesis of cancerous cells and the advancement of tumors. Recent evidence has substantiated the efficacy of directing attention towards transcription factors as a therapeutic modality in numerous cancer types [8–10]. The utilization of a strategy that focuses on the regulation of dysregulated transcription factors has been implemented in clinical settings and clinical trials. It is worth mentioning that blockers specifically focusing on ER or AR have shown efficacy in the management of breast and prostate tumors [11, 12]. CB-103, a specific small molecule inhibitor targeting the disruption of the Notch/RBPJ transcription factor complex, is presently under investigation in phase II clinical trials for its potential efficacy in treating drug-resistant cancers [13, 14]. However, the field of HCC lacks drugs or targets that are associated with transcription factors.

Early growth response 1 (EGR1), a zinc-finger transcription factor, is involved in important cellular processes including cell growth, metastasis, apoptosis, and DNA repair [15, 16]. Recently, Qin Tang et al. conducted sequencing assays and bioinformatic analysis, which revealed that EGR1 acts as a central regulator in HCC mouse models induced by DEN and HBX [17]. However, the role of EGR1 in hepatocarcinogenesis is still a subject of debate within the academic community. Existing literature suggests that EGR1 has the ability to act as both a suppressor and a promoter of tumors in HCC [18–22]. Nevertheless, it is crucial to acknowledge that these studies have not comprehensively examined the entire gene expression profile or conducted subsequent mechanistic analyses after manipulating EGR1, considering EGR1’s transcription factor nature. Therefore, additional research is necessary to clarify the exact function of EGR1 in the development of HCC and the molecular mechanisms involved.

In this study, the transcription factor target for HCC was identified as EGR1, which was found to be downregulated in HCC. In vitro experiments using EGR1 knockout or silence methods, along with EGR1 overexpression techniques, revealed that EGR1 had inhibitory impacts on the proliferation of HCC cells in vitro and the growth of xenografted tumors in vivo. Moreover, in a mouse model of HCC induced by DEN/CCL4, AAV-mediated EGR1 gene therapy exhibited the suppression of tumor growth and alleviation of liver injury. Furthermore, AAV-EGR1 was found to inhibit the growth of human hepatoma organoids in vitro. Additionally, our investigation revealed that EGR1 augmented the sensitivity of HCC cells and xenograft tumors to sorafenib. A mechanistic analysis, employing transcriptome sequencing, revealed that EGR1 engaged with the promoter region of phosphofructokinase-1, liver type (PFKL), resulting in the transcriptional suppression of PFKL expression and consequent inhibition of the glycolysis pathway. These results underscore the potential of EGR1 as a tumor suppressor gene in HCC and highlight its prospects for gene therapy.

## Materials and methods

### Clinical samples

The First Affiliated Hospital of Chongqing Medical University provided a total of 52 sets of HCC tissues along with their corresponding normal liver tissues. Twenty-six pairs of samples were utilized for Western blotting (WB) assay, while another twenty-six sets of samples were used for the reverse transcription quantitative polymerase chain reaction (RT-qPCR) assay. Subsequently, a total of five pairs of hepatocellular carcinoma tissues and their corresponding normal tissues were selected in a random manner for immunohistochemistry (IHC) staining.

### Cell culture

The normal liver cell line MIHA and liver cancer cell lines HepG2, MHCC97H, HCCLM3 and Hep3B were procured from Fudan University’s Zhongshan Hospital in Shanghai, China. Additionally, ATCC (located in Rockville, MD, USA) provided the cell lines PLC/PRF5 and Huh7. For the experiments, we used Dulbecco’s modified Eagle medium (DMEM, HyClone, USA), which was combined with 10% fetal bovine serum (FBS) and 1% penicillin/streptomycin (HyClone, USA) as additives. The cells were cultivated in an incubator with a 5% CO2 concentration at a temperature of 37 °C in a humidified setting.

### WB assay

Total protein was extracted from cells or tissues using RIPA lysis buffer (CWBIO, Jiangsu, China). WB assays were performed according to the guidelines provided by Abcam (https://www.abcam.cn/protocols/general-western-blot-protocol-2, accessed on 10 September 2021) utilizing a Bio-Rad gel analysis system (Bio-Rad, Hercules, CA, USA).WB bands were quantified using the ImageJ software(NIH, Bethesda, MD, USA). Supplementary Table S1 contained the listed commercial antibodies.

### Reverse transcription-quantitative polymerase chain reaction (RT-qPCR) of mRNA

With the TRIzol reagent (Thermo Fisher Scientific, Waltham, MA, USA), total RNA was extracted according to the recommended procedure by Abcam (https://www.abcam.cn/protocols/rna-isolation-protocol-cells-in-culture, accessed on 10 September 2021). Reverse transcriptions were performed in the subsequent procedures utilizing a Takara reverse kit (Takara, Kusatsu, Shiga, Japan). The Bimake SYBR Green qPCR master mix (Houston, TX, USA) was used for RT-qPCR on a Bio-Rad CFX connect real-time PCR detection system (Hercules, CA, USA). The Ct values of target genes were normalized to ACTB in the same sample, and gene expression analysis was performed using the 2-ΔΔCt method. Supplementary Table S1 contains the primers sequences for all genes.

### siRNAs and cell transfection

The siRNAs utilized in this study were acquired from TSINKE (Beijing, China) and comprised a negative control, EGR1#1 siRNA-F-CCAUGGACAACUACCCUAATT siRNA-R-UUAGGGUAGUUGUCCAUGGTT EGR1#2 siRNA-F- GCCUAGUG-AGCAUGACCAATT siRNA-R- UUGGUCAUGCUCACUAGGCTT. The transfection of siRNAs and plasmids was carried out using lipo8000 reagent (Beyotime, Shanghai, China).

### Plasmid DNA construction, lentivirus packaging and stable cell line generation

Prof. Yuan Hu (Key Laboratory of Molecular Biology on Infectious Diseases, Chongqing Medical University) provided the pCDH-CMV-MCS-EF1-copGFP-T2A-Puro plasmid. To achieve overexpression of EGR1 or PFKL, the coding sequences (CDs) of EGR1 or PFKL were inserted into the MCS domain of the expression plasmid. Prof. Ni Tang (Key Laboratory of Molecular Biology on Infectious Diseases, Chongqing Medical University) supplied the LentiCRISPR-v2, pMD2.G, and psPAX2 plasmids. The sgRNA sequences for CRISPR/Cas9-mediated gene editing targeting EGR1 or PFKL were acquired from the E-CRISP website (http://www.e-crisp.org/ECRISP/designcrispr.html, accessed on 12 August 2021). These sgRNAs were then incorporated into the lentiCRISPR-v2 plasmid. Subsequently, a total of 3 µg of lentiviral vectors were co-transfected into HEK293T cells along with 2 µg of psPAX2 and 1 µg of pMD2.G using the lipo8000 reagent (Beyotime, Shanghai, China). In order to establish stable monoclonal cell lines with EGR1 knockout, MHCC97H cells were subjected to infection with EGR1 sgRNA virus. Subsequently, the infected cells were isolated into individual clones within 96-well plates. The resulting single clones were then expanded and the knockouts were verified through western blot analysis and DNA sequencing.

### RNA sequencing

Cells were harvested for RNA sequencing assays using Trizol reagent (Invitrogen, Carlsbad, CA, USA), following the instructions provided in the manual. The cDNA libraries were then prepared and sequencing assays was conducted by Tsingke Corp. Laboratory (Beijing, China) utilizing the Illumina Novaseq 6000 platform (Tsingke, Beijing, China).

### Gene set enrichment analysis (GSEA)

The GSEA software (v4.1.0, accessible at www.broadinstitute.org/gsea, accessed on 6 June 2022) was utilized to perform gene set enrichment analysis (GSEA). The GSEA analysis employed hallmark gene sets obtained from the molecular signatures database (https://www.gsea-msigdb.org/gsea/msigdb/index.jsp, accessed on 15 June 2022).

### IncuCyte cell proliferation assay

IncuCyte live cell analysis system (Essen Bioscience, Ann Arbor, MI, USA) were used to evaluate cell proliferation. In 96-well plates, 1000–2000 cells were seeded per well and photographed every 24 h. After 120 h, cell proliferation was calculated based on phage-contrast images.

### EdU incorporation assay

The EdU incorporation assays were performed using an EdU cell proliferation kit containing Alexa Flour 555 (Epizyme, Shanghai, China) following the guidelines provided by the manufacturer. In summary, cells were seeded onto culture slides within 12-well plates. The plates were then exposed to EdU for a duration of 2 h and subsequently fixed in a 4% para-formaldehyde solution for 15 min. Following this, the cells were subjected to a 30-min click reaction and stained with Hoechst 33,342 for 10 min to visualize the nucleus. A laser scanning confocal microscope (Leica, Wetzlar, Germany) was employed to observe and capture images of the cells, while cell proliferation was assessed by determining the proportion of EdU positive cells.

### Colony formation assay

The cells were grown in 6-well dishes with a cell density of 1000 cells per well for a period of 14 days. After the 14-day duration, the cells were immobilized with 4% paraformaldehyde and subjected to crystal violet staining (Beyotime, Shanghai, China). The ability of the cell colony to form was subsequently evaluated based on the presence of colonies.

### Immunohistochemistry (IHC)

After cutting the paraffin-embedded tissues into 4 μm sections, they underwent deparaffinization using ethanol and xylene. Afterwards, the antigen was repaired by utilizing a pressure cooker. Next, using goat serum to block all sections, following the neutralization of endogenous peroxidase activity using 3% hydrogen peroxide. Subsequently, the sections were incubated with primary antibodies overnight at a temperature of 4 °C. Protein visualization was conducted by employing the ABC Peroxidase Staining Kit (Thermo Fisher Scientific, Waltham, MA, USA) and the DAB detection kit (ZhongShanJinQiao, Beijing, China) subsequent to secondary antibody incubation.

### Glucose uptake and extracellular lactate assays

Measurement of glucose uptake was performed using the Glucose Uptake-Glo™ Assay kit (Promega, WI, USA), while detection of extracellular lactate was accomplished using the Lactate Assay Kit-WST (Dojindo, Kumamoto, Japan), adhering to the guidelines in manual.

### Extracellular acidification rate (ECAR) assay

Extracellular acidification was measured by employing the extracellular acidification kit (BB-48311, Bestbio, China) following the instructions provided by the manufacturer. To detect ECAR, cells were initially seeded in 96-well black-bottom flat plates and allowed to incubate for 12 h prior to the experiment. Subsequently, the cells were incubated in a 37 °C incubator devoid of CO2 to eliminate any potential interference from CO2. Then cells were incubated with or without compounds (2-deoxy-D-glucose, 25 mM and oligomycin, 1.5 μM) and the pH sensitive BBcellProbe P61 probe was subsequently introduced to the cells, and the plates were analyzed using a microplate reader (BioTek Synergy H1, Winooski, VT, USA) at a temperature of 37 °C for a duration of 120 min, with measurements taken every 3 min (Ex488/Em580). The extracellular acidification rate (ECAR) was calculated as a ratio by dividing the difference between the final and initial fluorescence values by the time interval. Glycolysis was calculated as the discrepancy observed between the basal ECAR in the absence of any treatment and the ECAR measured during the incubation period of 2-deoxy-D-glucose (2-DG). Glycolytic capacity was calculated as the discrepancy observed between the ECAR measured during the incubation period of oligomycin and the ECAR measured during the incubation period of 2-DG.

### ATP assay

The ATP levels were detected by the ATP regents using a ADP/ATP ratio assay kit (Dojindo, Kumamoto, Japan) according to the commercial protocol.

### Chromatin immunoprecipitation assay (ChIP)

The ChIP assay kit (Wanleibio, Liaoning, China) was utilized to conduct the chromatin immunoprecipitation assay, following the guidelines provided by the manufacturer. Initially, cells were fixed with 16% paraformaldehyde (CST, MA, USA) at a concentration of 1% to facilitate the crosslinking of DNA and proteins. Subsequently, genomic DNA was fragmented through sonication (10 s on, 30 s off, 30% amplitude, 12 cycles, QSonica Q800R3 Sonicator, CT, USA) and then immunoprecipitated using either the EGR1 ChIP grade antibody or a normal rabbit IgG antibody. On the following day, the immunoprecipitation complexes were subjected to incubation with protein A + G beads for a duration of 2 h at a temperature of 4 °C. Subsequently, the DNA–protein complex was subjected to de-crosslinking at a temperature of 65 °C overnight. On the third day, DNA was retrieved utilizing a PCR purification kit (Wanleibio, Liaoning, China) and subsequently subjected to analysis through qRT-PCR assays. Supplementary Table S1 contains the primer sequences.

### Dual luciferase reporter assay

The cells were placed in 24-well dishes and co-transfected with the suitable reporter plasmids. Following a 2-day period of plasmid DNA transfection, the cells underwent examination utilizing a dual luciferase reporter gene assay kit (Yeasen biotechnology, Shanghai, China) as per the guidelines provided by the manufacturer. The Glomax multi-detection system (Promega, WI, USA) was utilized to measure the activity of firefly luciferase and renilla luciferase.

### Human hepatoma organoids assays

The generation and analysis of human liver cancer organoids were conducted by Hangzhou Hunter Biotechnology Co., Ltd. In brief, hepatoma organoids were established and cultured using a hepatocellular carcinoma organoid medium (HTC-HC01-100, NEWHUNTER, China) supplemented with 80% Matrigel (Corning, New York, NY, USA) in 24-well plates. Subsequently, the organoids were transfected with 200 ul of virus-containing 1.0 E + 11 AAV2/8 viral genome particles for a duration of 24 h. Following that, the organoids were gathered and reseeded in 24-well plates. The growth of the organoids was then observed and documented using a Nikon Eclipse Ti-E inverted fluorescence microscope (Nikon TS2R-FL, Nikon instruments Inc., USA) on the first, third, and fifth days of incubation, respectively.

### Animal models

For the xenograft tumor assays, nude mice were obtained from ENSIWEIER Corporation (Chongqing, China). A total of 5 × 106 MHCC97H cells in 100 ul PBS or 5 × 106 HepG2 cells mixed with PBS and Matrigel solution (1:1 ratio, ABW Matrigengel, Shanghai, China) were injected subcutaneously into the right flanks of the nude mice (*n* = 5 per group). The mice were observed every three days. In the experiments involving the combination of AAV2/8-EGR1 with sorafenib for the treatment of subcutaneous tumors, AAV2/8-EGR1 was obtained from OBiO Technology Corp., Ltd (Shanghai, China). Once the tumor dimensions reached 5 mm × 5 mm (length × width), an intratumoral injection of 100 μl of virus containing 8.0 E + 10 AAV2/8 viral genome particles and 10 mg/kg sorafenib (administered twice a week for a duration of 2 weeks) was administered.

A mouse model of HCC was established using 4-week-old C57 mice. The primary HCC was induced by administering diethyl nitrosamine (DEN) at a dosage of 75 mg/kg, followed by an additional dose of 25 mg/kg two weeks later. Carbon tetrachloride (CCl4) was then administered twice weekly at a dosage of 1 ml/kg for a duration of 32 weeks. Starting from the 20th week, the mice received tail vein injections of 200 ul of virus-containing 2.0 E + 11 AAV2/8 viral genome particles. At the conclusion of the 36th week, the mice were euthanized, and their livers and serum were collected for immunohistochemical staining and analysis of serum markers.

### Serum markers analysis

ALT and AST levels were measured using an ALT/GPT and AST/GOT assay kit (Nanjingjiancheng, Nanjing, China), respectively, following the established protocols.

### Statistical analysis

GraphPad Prism 8.3.0 was utilized for the purpose of conducting the data analysis. The values are presented as the mean ± standard deviation. The differences between two groups were assessed using unpaired or paired Student’s t-test. The examination of the relationships between two variables utilized Pearson’s correlation coefficient. The statistical significance was established by considering *P < 0.05, **P < 0.01, ***P < 0.001, ****P < 0.0001, or ns (indicating no significance).

## Results

### EGR1 identified as a transcriptional factor target in HCC and EGR1 was downregulated in HCC

**Fig. 1:**
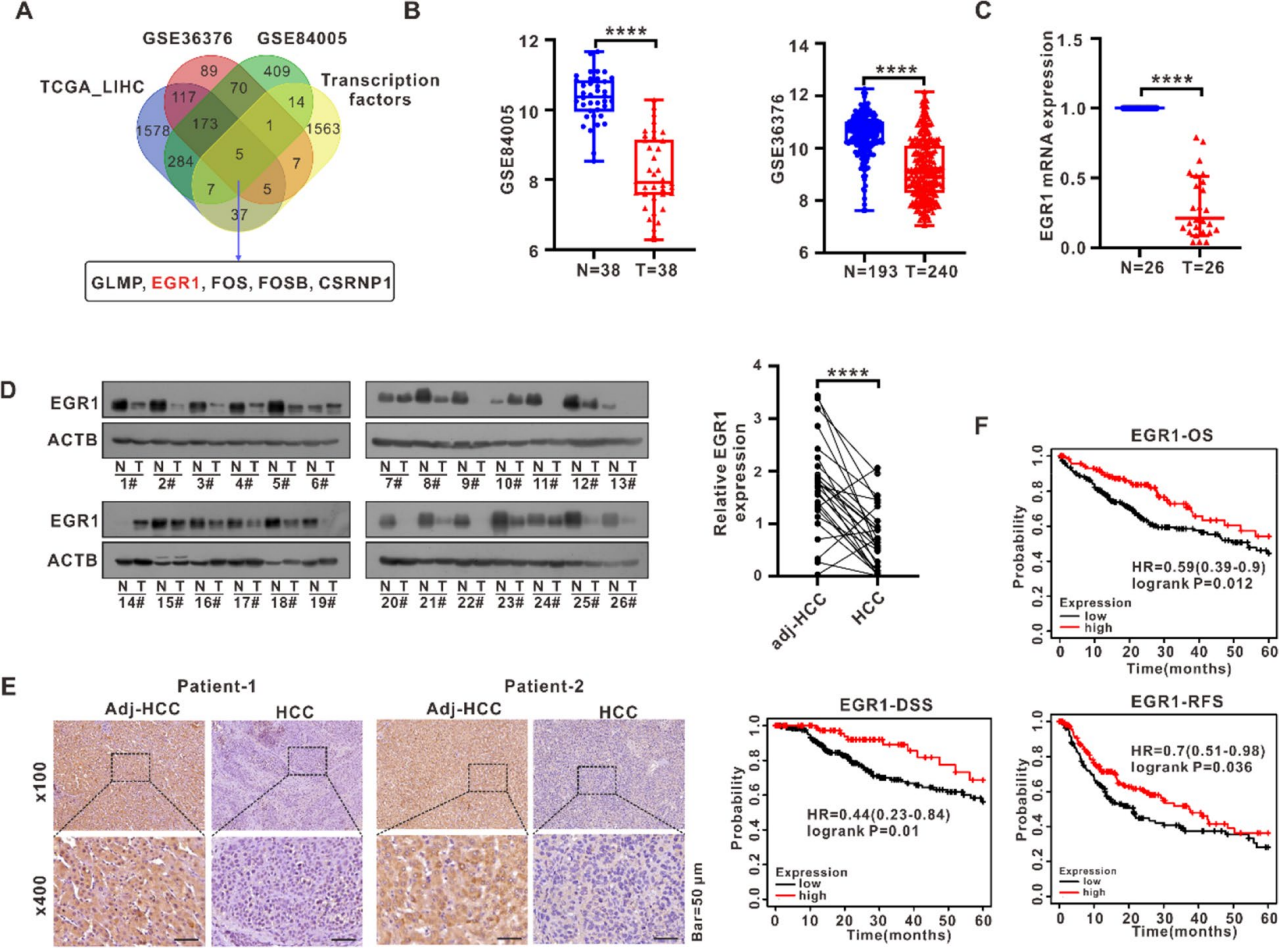
EGR1 identified as a transcriptional factor target in HCC and EGR1 was downregulated in HCC. **A** Five candidate transcription factors targets, including EGR1, were identified based on the intersection of differential genes from the TCGA_LIHC dataset, two HCC GEO datasets, and a transcription factor gene set. **B** The mRNA expression of EGR1 was analyzed in two HCC GEO datasets (GSE36376, GSE84005). **C** The mRNA expression of EGR1 was examined in 26 pairs of HCC clinical samples. **D** The protein expression of EGR1 was investigated in 26 pairs of HCC clinical samples, and a scatter plot illustrating the relative quantification of EGR1 protein expression is presented. **E** The protein expression of EGR1 was confirmed in five pairs of HCC clinical samples using immunohistochemistry staining. **F** Kaplan–Meier survival analysis of the OS, DSS and RFS rates of patients expressing high or low levels of EGR1 using the Kaplan–Meier plotter survival analysis tool (https://kmplot.com/analysis/). The samples were assigned into EGR1 high/low cohorts by the best available cut-off value. ****P < 0.0001

In order to identify transcriptional targets in HCC, we obtained a total of 2207 differentially expressed genes (DEGs) from the TCGA_LIHC (The Cancer Genome Atlas_ Liver hepatocellular carcinoma) dataset, 467 and 963 DEGs from two HCC datasets (GSE36376, GSE84005) respectively, and 1693 human transcription factors from the humanTFs database (http://humantfs.ccbr.utoronto.ca/allTFs.php, accessed on 10 May 2021). The 5 genes that were found to be common among these four gene sets were considered as potential transcription factors targets toward HCC (Fig. 1A). Based on the findings of sequencing and bioinformatic research, a recent study confirmed that EGR1, ATF3 and KLF4 were the core TF (transcription factor) regulators in HCC [17]. However, the role of EGR1 in HCC is ambiguous. For the purpose of this study, our focus was on investigating the role of EGR1 through subsequent experiments. EGR1, a transcription factor of considerable significance, has been documented to play pivotal roles in numerous types of cancer. Then we found EGR1 mRNA and protein expression in HCC both showed low levels among all cancer types based on TCGA and HPA (Human Protein Atlas) databases (Fig. S1A, B). In order to further investigate EGR1 expression in HCC, EGR1 expression was examined across two HCC GEO datasets (GSE36376, GSE84005), confirming its downregulation in HCC (Fig. 1B). Following this, the mRNA expression of EGR1 was examined in twenty-six pairs of HCC tissue samples and their corresponding neighboring normal tissues (Fig. 1C). Additionally, the protein expression of EGR1 was assessed in another twenty-six pairs of HCC tissue samples and their corresponding neighboring normal tissues (Fig. 1D). The results indicated a notable decrease in the levels of EGR1 mRNA and protein within the HCC tissues when compared to the surrounding normal tissues. Subsequently, a random subset of five pairs of HCC tissues underwent immunohistochemical analysis to evaluate the expression of EGR1 protein. The results unequivocally validated the downregulation of EGR1 in HCC (Fig. 1E). To investigate the impact of EGR1 levels on patient outcomes, the Kaplan–Meier method was employed to conduct a survival analysis on the overall survival (OS), disease-specific survival (DSS), and recurrence-free survival (RFS) rates among patients expressing high or low levels of EGR1 and the results showed that high EGR1 expression associated with better survival (Fig. 1F).

### EGR1 downregulation prompted HCC cells proliferation in vitro and facilitated tumor growth in vivo

**Fig. 2:**
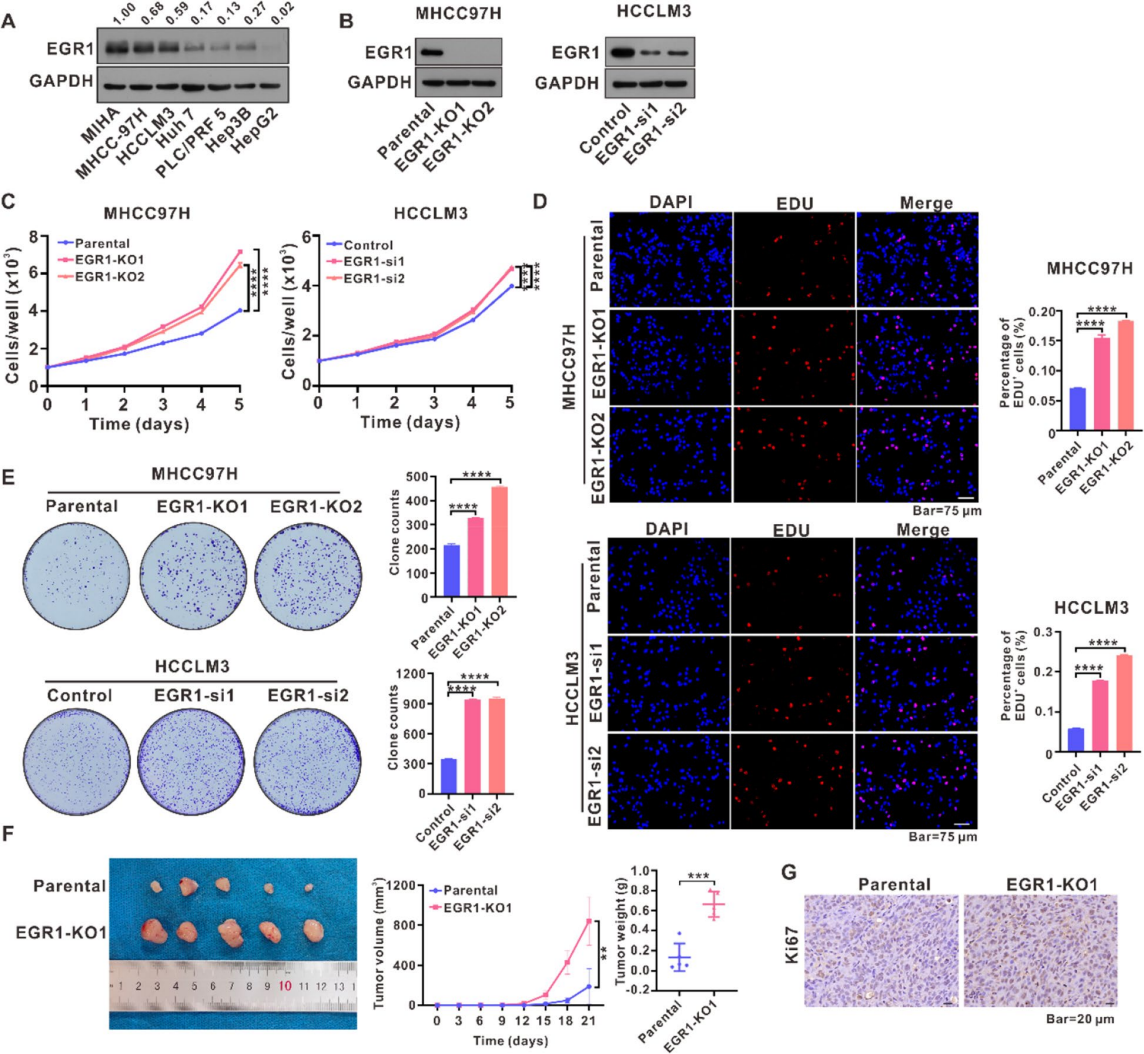
EGR1 downregulation prompted HCC cells proliferation in vitro and facilitated tumor growth in vivo. **A** We performed WB assays to evaluate the presence of EGR1 in normal liver cell line and HCC cell lines. **B** The protein expression of EGR1 was observed in MHCC97H and HCCLM3 cells following the knockout or silencing of EGR1 using WB assays. **C** The IncuCyte zoom cell proliferation assays were used to assess the effect of EGR1 knockout or silencing on the growth of HCC cells. **D** To validate the influence of EGR1 knockout or silencing on the growth of HCC cells, EDU incorporation assays were performed. **E** The ability of HCC cells to form colonies was evaluated by conducting colony formation assays following the knockout or silencing of EGR1. **F** Xenograft tumor assays were conducted on parental and EGR1 knockout MHCC97H cells in order to examine the impact of EGR1 knockout on the growth of xenograft tumors. Tumor volume was assessed at three-day intervals, and after 21 days, the tumors were gathered and measured in terms of weight. **G** Ki67 staining of the xenograft tumors was employed to validate the influence of EGR1 knockout on the growth of xenograft tumors. **P < 0.01, ***P < 0.001, ****P < 0.0001

To investigate the role of EGR1 in HCC, we conducted an analysis of EGR1 protein expression in both normal liver cell line and HCC cell lines. Our findings revealed that HCC cell lines exhibited a significantly lower protein and mRNA expression of EGR1 compared to normal liver cell line (MIHA) (Fig. 2A and Fig. S2A). Subsequently, we utilized CRISPR/Cas9 technology to generate two single clones of EGR1 knockout (KO) cell lines in MHCC97H cells, while EGR1 expression was silenced in HCCLM3 cells using EGR1 siRNAs (Fig. 2B). We then proceeded to investigate the impact of EGR1 downregulation on HCC growth through various in vitro assays. The IncuCyte cell proliferation assays revealed that the downregulation of EGR1 stimulated the proliferation of HCC cells (Fig. 2C). Subsequently, the incorporation of EDU was employed to monitor cell proliferation, and the quantification was determined by the percentage of EDU-positive (EDU +) cells. The findings demonstrated that the downregulation of EGR1 increased the proportion of cells incorporating EDU (Fig. 2D). Colony formation assays were performed to evaluate the effect of reducing EGR1 on the ability to form colonies. The results indicated that the downregulation of EGR1 improved the capacity of HCC cells to establish colonies (Fig. 2E). In order to investigate the effect of reducing EGR1 on the growth of HCC tumors, we implanted both the parental MHCC97H cells and the MHCC97H cells with EGR1 knockout into nude mice for an in vivo examination. Subsequently, the tumor sizes were assessed at three-day intervals. Following a period of twenty-one days, the analysis of tumor growth curve, tumor sizes, tumor weight and Ki67 staining demonstrated that EGR1 downregulation significantly stimulated tumor growth in the in vivo setting (Fig. 2F, G).

### EGR1 inhibited HCC cells proliferation in vitro and attenuated tumor growth in vivo

**Fig. 3:**
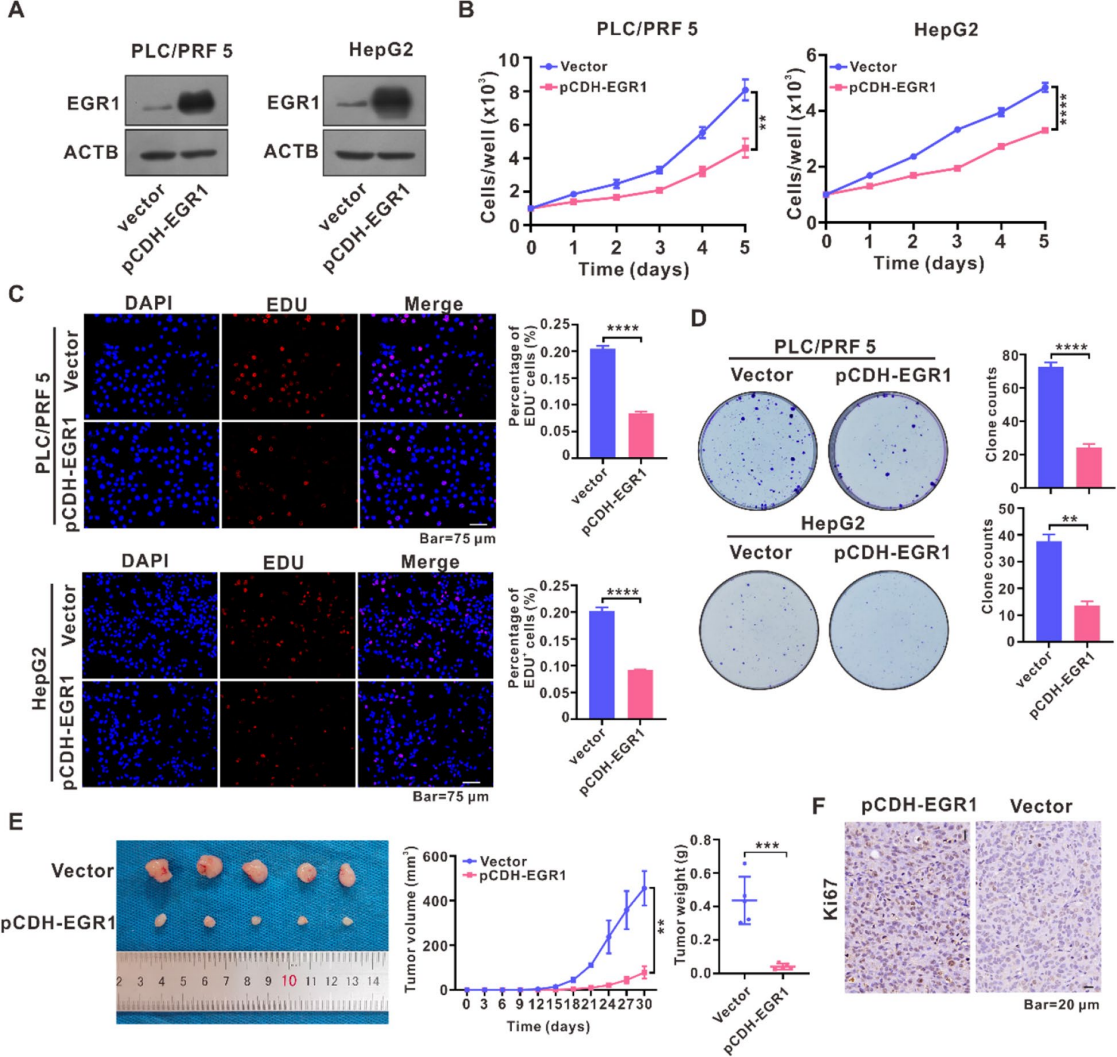
EGR1 inhibited HCC cells proliferation in vitro and attenuated tumor growth in vivo. **A** Western blot assays revealed the presence of EGR1 protein in PLC/PRF5 and HepG2 cells after EGR1 overexpression. **B** The impact of EGR1 on cell proliferation was assessed using IncuCyte zoom cell proliferation assays. **C** EDU incorporation assays were performed to evaluate the impact of EGR1 on the growth of HCC cells. **D** Colony formation experiments were conducted to assess the impact of EGR1 on the ability to form colonies. **E** HepG2 cells were utilized to investigate the effect of EGR1 on the growth of xenograft tumors. Tumor volume was measured every three days, and after 30 days, the tumors were harvested and weighed. **F** Ki67 staining of the xenograft tumors was employed to validate the influence of EGR1 overexpression on the growth of xenograft tumors. **P < 0.01, ***P < 0.001, ****P < 0.0001

To further explore the function of EGR1 in HCC, we conducted overexpression experiments of EGR1 in PLC/PRF5 and HepG2 cells (Fig. 3A). Afterwards, multiple in vitro tests were performed to evaluate the influence of EGR1 on the growth of HCC cells. The IncuCyte cell proliferation assays demonstrated that EGR1 exerted a suppressive impact on the growth of PLC/PRF5 and HepG2 cells (Fig. 3B). Furthermore, the EDU incorporation assays revealed a decrease in the proportion of EDU-incorporated PLC/PRF5 and HepG2 cells in response to EGR1 (Fig. 3C). Additionally, the colony formation assays showed a decrease in the quantity of colonies formed by PLC/PRF5 and HepG2 cells upon EGR1 expression (Fig. 3D). Nude mice were injected with HepG2 cells and the size of the tumor was monitored every three days. After thirty days, the tumor samples were harvested and EGR1 attenuated tumor growth (Fig. 3E, F).

### EGR1 suppressed aerobic glycolysis in HCC cells

**Fig. 4:**
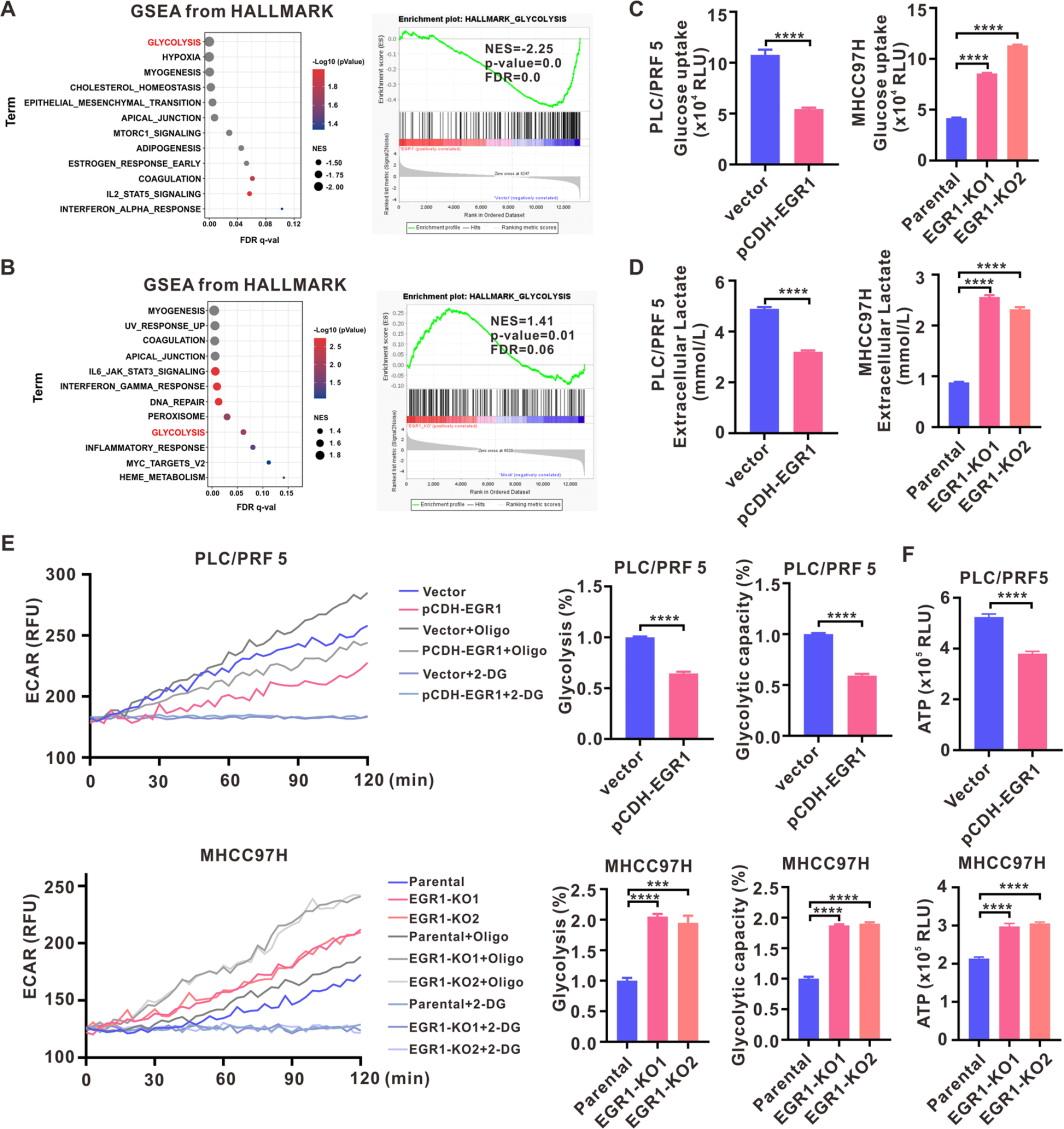
EGR1 suppressed aerobic glycolysis in HCC cells. **A** The GSEA analysis using hallmark gene sets in EGR1 overexpressing PLC/PRF5 cells and the result showed that EGR1 overexpression downregulated glycolysis pathway. **B** The GSEA analysis using hallmark gene sets in EGR1 knockout MHCC97H cells and the result revealed that EGR1 knockout upregulated glycolysis pathway. **C**, **D**, **E**, **F** The impact of EGR1 on glucose uptake, extracellular lactate levels, extracellular acidification rates (ECARs), and ATP levels was investigated. ***P < 0.001, ****P < 0.0001

To clarify the molecular mechanism that explains the suppressive impact of EGR1 on HCC growth, we performed RNA-seq experiments on PLC/PRF5 cells with EGR1 overexpression. The results of Gene Set Enrichment Analysis (GSEA) demonstrated a significant downregulation of glycolysis pathways by EGR1 (Fig. 4A). Aerobic glycolysis, referred to as the Warburg phenomenon, is widely acknowledged as a prominent hallmark of cancer and significantly contributes to the advancement of the disease. Subsequently, we conducted RNA-seq experiments on MHCC97H cells with a knockout of EGR1, and the GSEA analysis revealed that the absence of EGR1 resulted in an upregulation of glycolysis pathway (Fig. 4B). Glycolysis serves as the predominant source of ATP in many tumor cells. We evaluated the metabolic pathway dependency in four HCC cells and the results showed that HCC cells are highly dependent on glycolysis for the generation of ATP in our experimental conditions (Fig. S3A). Glycolysis is distinguished by heightened glucose consumption and augmented lactate production, which arises as a result of glucose catabolism. In order to confirm the influence of EGR1 on glycolysis in HCC cells, we assessed glucose uptake and lactate levels in both EGR1-overexpressing PLC/PRF5 cells and EGR1 knockout MHCC97H cells. The findings demonstrated that EGR1 overexpression led to a decrease in glucose uptake and extracellular lactate levels in PLC/PRF5 cells, whereas EGR1 knockout resulted in an increase in glucose uptake and extracellular lactate levels in MHCC97H cells (Fig. 4C, D). Then the ECAR assays showed that EGR1 overexpression significantly reduced glycolysis and glycolytic capacity, whereas EGR1 knockout led to a rise in glycolysis and glycolytic capacity (Fig. 4E). Additionally, we detected the ATP levels and the results showed EGR1 overexpression decreased the ATP levels in HCC cells whereas EGR1 knockout increased ATP levels (Fig. 4F). The aforementioned observations offer proof that EGR1 inhibited aerobic glycolysis in HCC.

### EGR1 transcriptionally downregulated PFKL in HCC cells

**Fig. 5:**
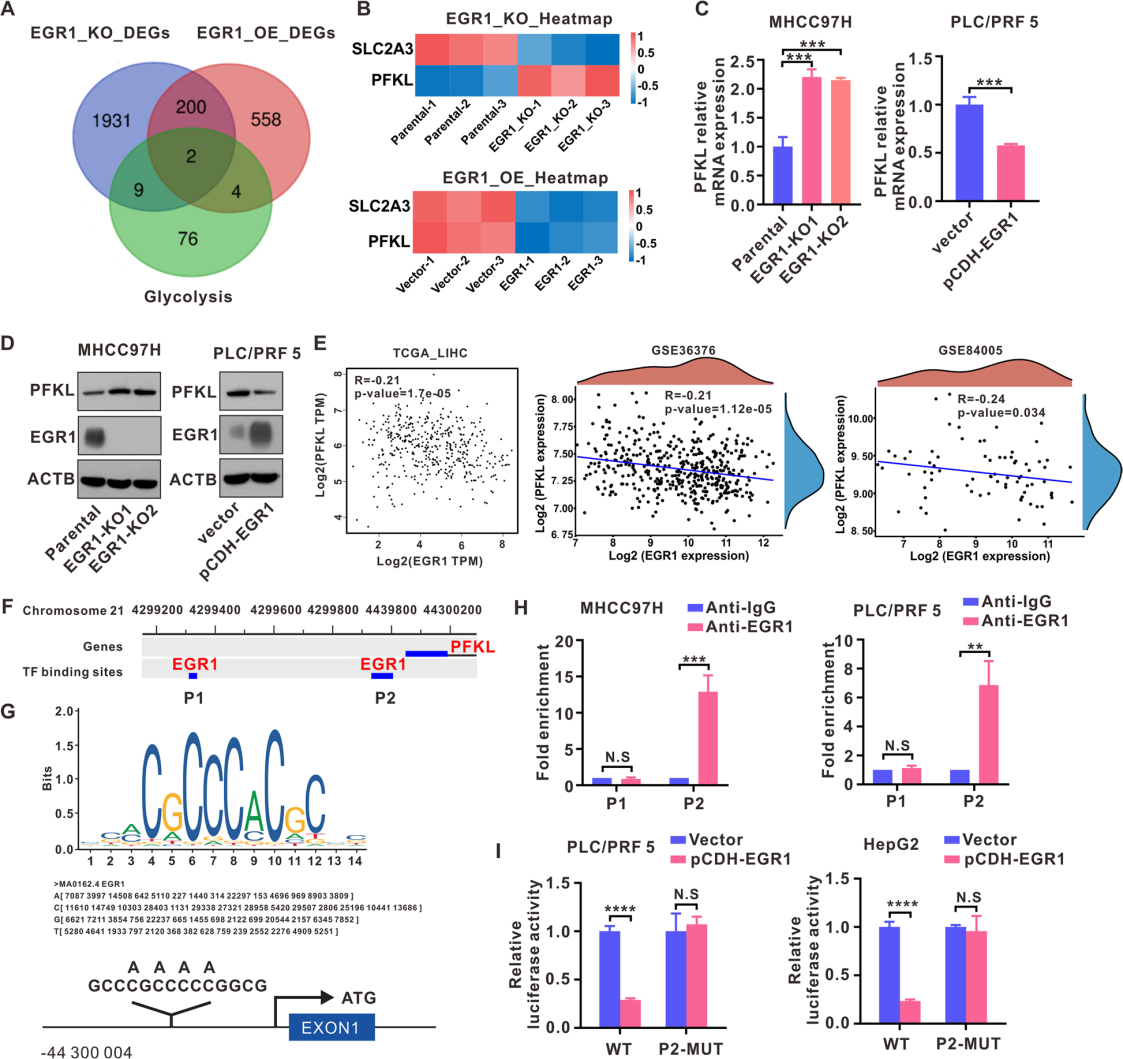
EGR1 transcriptionally downregulated PFKL in HCC cells. **A** The intersection of genes among EGR1 knockout differential genes, EGR1 overexpression differential genes, and glycolysis-related genes was examined. **B** The mRNA expression of SLC2A3 and PFKL in EGR1 knockout and EGR1 overexpression HCC cells was analyzed using transcriptome sequencing data. **C** The levels of PFKL mRNA were measured in MHCC97H and PLC/PRF5 cells using RT-qPCR. **D** The presence of PFKL protein was determined in EGR1 knockout MHCC97H cells and EGR1 overexpressing PLC/PRF5 cells. **E** A correlation analysis was performed to evaluate the association between EGR1 and PFKL using data from the TCGA database and two GEO datasets. **F** The GTRD database was used to predict the binding sites of EGR1 in the promoter region of PFKL. **G** The potential EGR1 binding site in JASPAR is represented by the sequence logo in the top panel, whereas the bottom panel illustrates the mutant PFKL promoter sites. **H** CHIP assays were conducted in MHCC97H and PLC/PRF5 cells. **I** Dual luciferase reporter assays provided evidence of the binding of EGR1 in P2 of PFKL. **P < 0.01, ***P < 0.001, ****P < 0.0001, N.S (not significant)

In order to further elucidate the underlying molecular mechanism by which EGR1 suppresses aerobic glycolysis, we calculated the intersection of EGR1 overexpression DEGs, EGR1 knockout DEGs, and glycolysis-related genes (Fig. 5A). Our analysis revealed that PFKL, a key enzyme in the glycolysis process, was upregulated after EGR1 knockout, while PFKL was downregulated after EGR1 overexpression (Fig. 5B). Following that, we performed RT-qPCR and western blot experiments to validate the alterations in PFKL mRNA and protein levels in MHCC97H and PLC/PRF5 cells (Fig. 5C, D). According to TCGA_LIHC dataset and two GEO datasets (GSE36376, GSE84005), PFKL mRNA expression and EGR1 showed a clear inverse correlation (Fig. 5E). In GTRD (gene transcription regulation database) database, a gene transcription regulation database based on CHIP-seq data, we predicted putative EGR1 binding sequences within the promoter region of PFKL and identified two potential binding sequences, part 1 (P1) and part 2 (P2) (Fig. 5F). JASPAR was used to obtain EGR1 binding motif (Fig. 5G). The CHIP assay results indicated a significant enrichment of EGR1 in the P2 region of the PFKL promoter (Fig. 5H). Following this, the dual luciferase reporter assay revealed that EGR1 had a repressive impact on the luciferase activity of the original PFKL promoter, but it had no influence on the luciferase activity of the P2 mutant in the PFKL promoter (Fig. 5I). The aforementioned experimental findings collectively indicate that PFKL is subject to direct transcriptional repression by EGR1 in HCC.

### EGR1 inhibited HCC cells proliferation by downregulating PFKL-mediated aerobic glycolysis

**Fig. 6:**
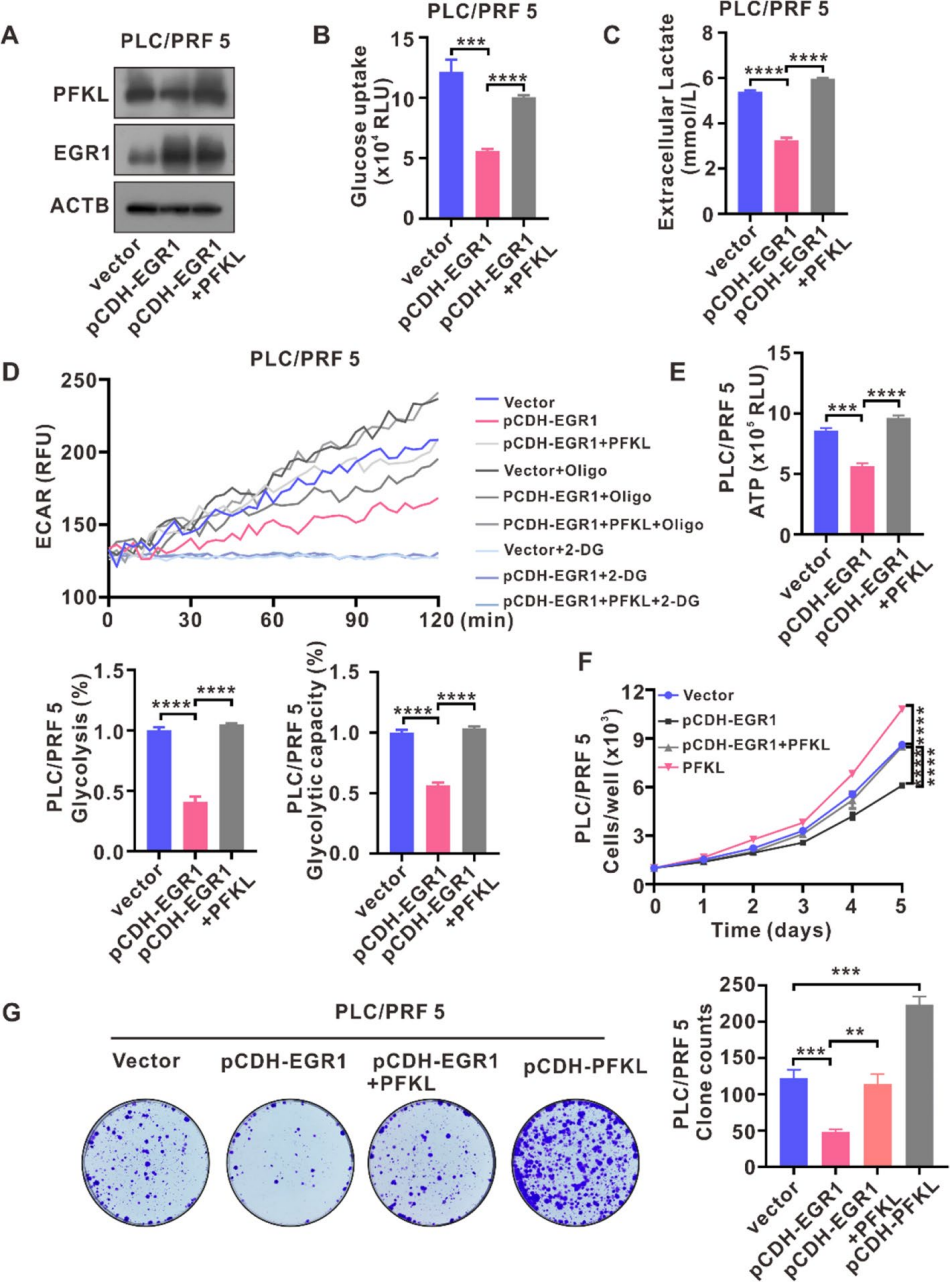
EGR1 inhibited HCC cells proliferation by downregulating PFKL-mediated aerobic glycolysis. **A** The WB analysis was conducted on PFKL and EGR1 in EGR1 overexpressing PLC/PRF5 cells after PFKL restoration. **B**, **C**, **D**, **E**, **F**, **G** PFKL restoration was observed to reverse the inhibitory effects of EGR1 on glucose uptake, extracellular lactate levels, glycolysis and glycolytic capacity, ATP levels, cell proliferation and colony formation capacity. **P < 0.01, ***P < 0.001, ****P < 0.0001

Further investigating the role of PFKL downregulation in the tumor suppressive activity of EGR1 in HCC, we overexpressed PFKL in EGR1-overexpressing PLC/PRF5 cells (Fig. 6A). Through measurements of glucose uptake, extracellular lactate levels, ECAR and ATP levels, our findings indicate that restoration of PFKL expression increased glucose uptake, extracellular lactate levels, glycolysis, glycolytic capacity, and ATP levels in EGR1 overexpressing PLC/PRF5 cells (Fig. 6B-E). The Incucyte zoom cell proliferation assays demonstrated that PFKL overexpression in PLC/PRF5 cells effectively abrogated the repression of proliferation mediated by EGR1 (Fig. 6F). Accordingly, the colony formation assays demonstrated similar trends in PLC/PRF5 cells (Fig. 6G). Subsequently, we used PFKL sgRNA to silence PFKL expression in EGR1 knockout MHCC97H cells (Fig. S4A). Through detections of glucose uptake, extracellular lactate levels, ECAR and ATP levels, our results showed that the increased glucose uptake, extracellular lactate levels, glycolysis, glycolytic capacity, and ATP levels in EGR1 knockout MHCC97H cells were blocked by PFKL silence (Fig. S4B-E). The Incucyte zoom cell proliferation assays and colony formation assays demonstrated that the increased cell proliferation and colony formation capacity induced by EGR1 knockout was blocked by PFKL silence in MHCC97H cells (Fig. S4F, G). These findings collectively suggest that EGR1 suppresses aerobic glycolysis and HCC proliferation by downregulating PFKL expression.

### AAV-EGR1 inhibited HCC in a DEN/CCL4 driven mouse model of HCC in vivo and inhibited human hepatoma organoid growth in vitro

**Fig. 7:**
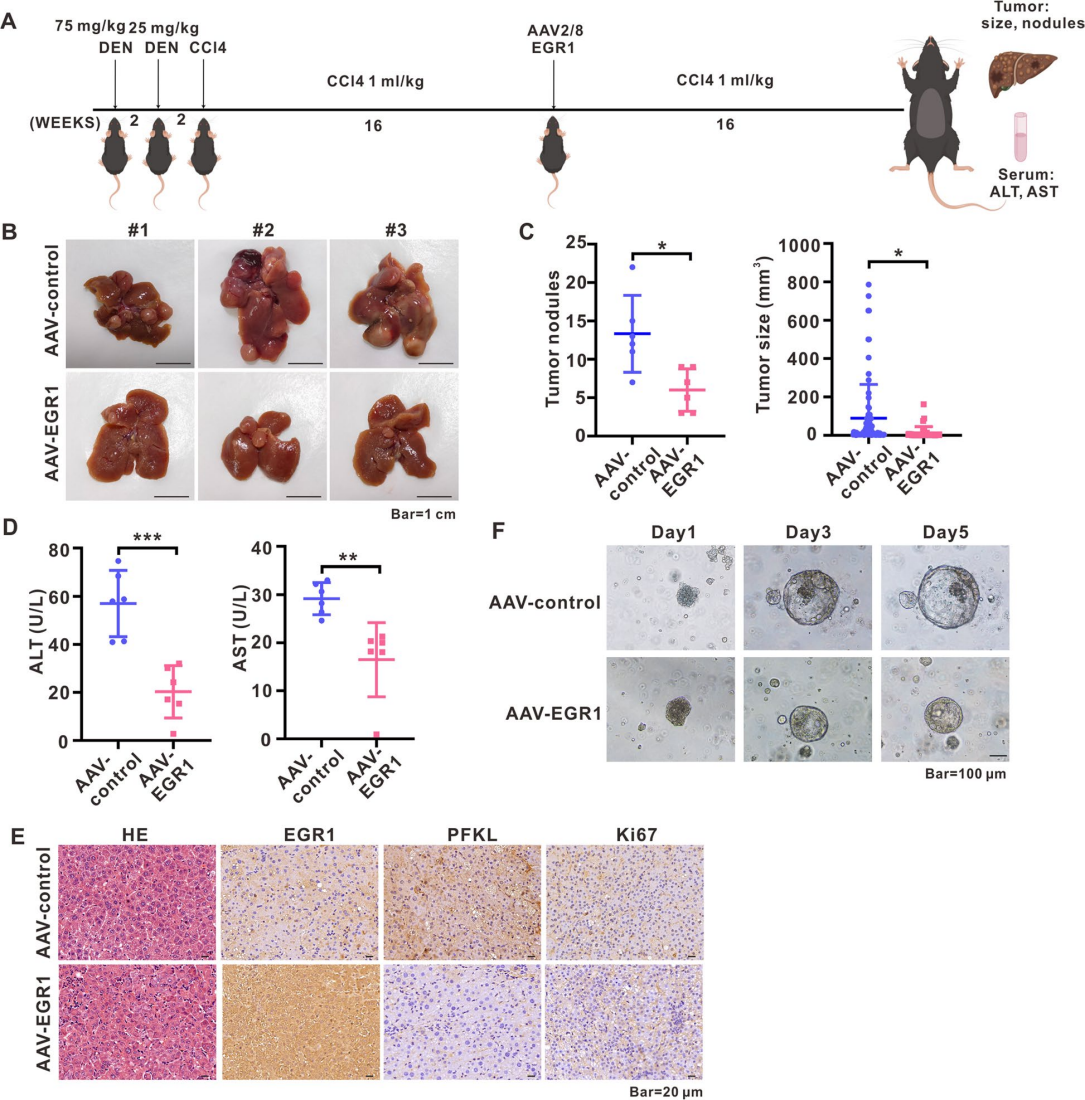
AAV-EGR1 inhibited HCC in a DEN/CCL4 driven mouse model of HCC in vivo and inhibited human hepatoma organoid growth in vitro. **A** DEN/CCl4 induced mice with HCC were treated with AAV-EGR1 in vivo to examine the impact of AAV-EGR1 on tumor development. **B** The anti-tumor effect of AAV-EGR1 was observed in comparison with AAV-control through typical images of tumor-bearing mice livers; the number of mice in each treatment group was *n* = 6. **C** Tumor nodules were quantified and tumor sizes were measured upon liver harvest. **D** The serum of mice was tested for the levels of ALT and AST. **E** The staining of HE, EGR1, PFKL, and Ki67 in mice livers was presented. **F** Representative images of human hepatoma organoid demonstrated the anti-tumor effect of AAV-EGR1 in comparison with AAV-control. *P < 0.05, **P < 0.01, ***P < 0.001

In order to conduct a more comprehensive examination of the potential inhibitory effects of EGR1 gene therapy on HCC progression, we established a mouse model of HCC using DEN/CCL4 induction (Fig. 7A). Subsequently, liver samples and serum specimens were obtained after 16 weeks of AAV-EGR1 gene therapy for further analysis. The statistical analysis of tumor nodules and tumor sizes revealed that AAV-EGR1 treatment significantly attenuated the growth of HCC tumors (Fig. 7B, C). Furthermore, the serum specimens were assessed for the levels of alanine aminotransferase (ALT) and aspartate aminotransferase (AST), which act as indicators of liver damage. The results indicated that AAV-EGR1 gene therapy resulted in a reduction of ALT and AST levels (Fig. 7D). The utilization of Hematoxylin and eosin (HE), EGR1, PFKL, and Ki67 staining on liver samples provided confirmation that AAV-EGR1 intervention resulted in a decrease in PFKL expression and a reduction in the proportion of Ki67 positive cells (Fig. 7E). Additionally, the implementation of in vitro human hepatoma organoid models further validated the inhibitory effects of AAV-EGR1 on human hepatoma organoid growth (Fig. 7F).

### EGR1 enhanced the sensitivity of HCC cells and xenografted tumors to sorafenib

**Fig. 8:**
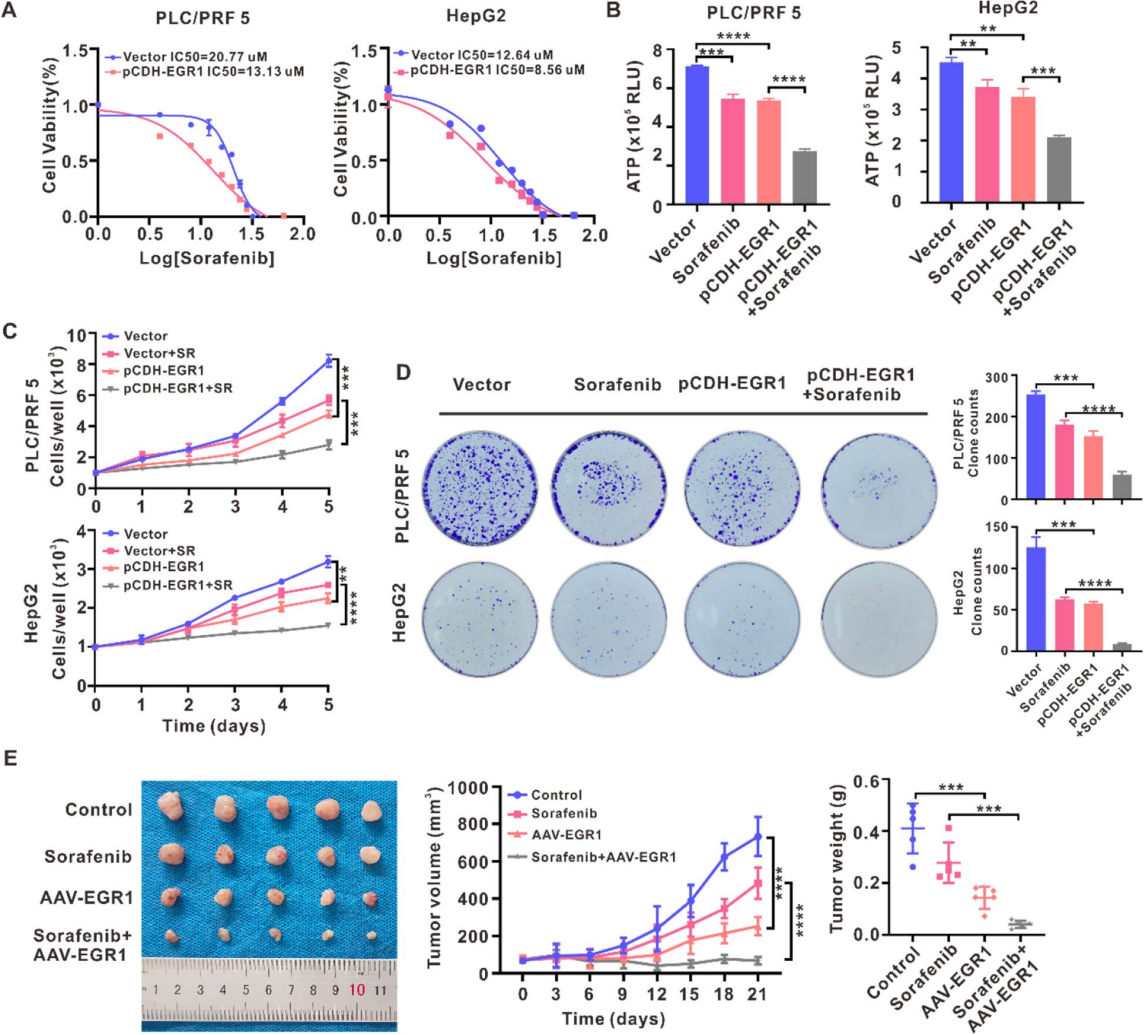
EGR1 enhanced the sensitivity of HCC cells and xenografted tumors to sorafenib. **A** The IC50 values of sorafenib was detected in PLC/PRF5 and HepG2 cells after EGR1 overexpression. **B** The ATP levels in HCC cells after EGR1 overexpression, sorafenib treatment and the combinational treatment were detected. **C** IncuCyte zoom cell proliferation assays were performed to evaluate the combined effect of EGR1 and sorafenib on PLC/PRF5 and HepG2 cells. **D** The impact of the combination of EGR1 and sorafenib on the capacity of HCC cells to form colonies was examined through colony formation assays. **E** Xenograft tumor experiments were conducted to assess the influence of AAV-EGR1 in conjunction with sorafenib on the growth of HCC tumors. Tumor dimensions were measured at three-day intervals, and tumor weight was determined upon tumor harvest. **P < 0.01, ***P < 0.001, ****P < 0.0001

Sorafenib represents the initial choice of systematic medication for patients with advanced HCC. Nevertheless, the therapeutic effectiveness of sorafenib is constrained by the development of acquired resistance. Luyuan Ma et al. and Ruize Gao et al. found that the upregulation of the aerobic glycolysis pathway contributes to the acquired resistance of sorafenib in HCC [23, 24]. The findings of our study have provided evidence for the association between EGR1 downregulation and the induction of aerobic glycolysis. In order to explore the potential impact of EGR1 downregulation on the efficacy of sorafenib in HCC, we utilized the genomics of drug sensitivity in cancer (GDSC) database to predict IC50 values of sorafenib based on EGR1 expression. The results demonstrated a notable rise in the IC50 values of sorafenib among HCC patients with lower levels of EGR1 expression (Fig. S5A). Furthermore, an analysis of a GEO dataset demonstrated a downregulation of EGR1 expression in sorafenib-resistant HepG2 cells (Fig. S5B). Intriguingly, our investigation revealed a decrease of EGR1 expression in surviving HCC cells subsequent to treatment with a high concentration of sorafenib (Fig. S5C, D). The aforementioned findings provide compelling evidence that the downregulation of EGR1 expression could potentially play a role in the emergence of sorafenib resistance in HCC. Subsequently, we proceeded to assess the IC50 values in HCC cells after alteration of EGR1 expression. The results demonstrated that EGR1 downregulation led to an elevation in IC50 values of sorafenib in MHCC97H and HCCLM3 cells, whereas glycolysis inhibitor 2-DG abrogated the increased IC50 values of sorafenib in EGR1 knockout MHCC97H cells and EGR1 silencing HCCLM3 cells (Fig. S5E). Furthermore, EGR1 knockout or silence promoted HCC cells proliferation and colony formation in the context of sorafenib meanwhile the increased cell proliferation and colony formation capacity were abrogated by 2-DG (Fig. S5F, G). Conversely, EGR1 overexpression resulted in a reduction of sorafenib IC50 values in PLC/PRF5 and HepG2 cells (Fig. 8A). Tumor growth requires a massive amount of ATP as an energy supply. Previous research has reported that sorafenib treatment can inhibit ATP production in HCC cells [25], and the combination of sorafenib with 2-DG synergistically inhibits ATP production and HCC cell proliferation [26]. To investigate the combinational effect of sorafenib and EGR1 overexpression on HCC growth, we detected the ATP levels in HCC cells after EGR1 overexpression, sorafenib treatment and the combinational treatment, the results showed single EGR1 overexpression or sorafenib treatment decreased ATP levels and the combinational treatment further reduced ATP production (Fig. 8B). Then, the IncuCyte cell proliferation assays and colony formation assays further revealed that EGR1 indeed augmented the inhibitory effect of sorafenib on cell proliferation and colony formation capacity of PLC/PRF5 and HepG2 cells (Fig. 8C, D). Additionally, an in vivo animal model confirmed the efficacy of AAV-EGR1 in reducing HCC tumor growth and improving the therapeutic outcomes of sorafenib (Fig. 8E). These findings collectively suggest that the combination of sorafenib and EGR1 gene therapy may provided benefit compared to single sorafenib treatment in HCC patients.

## Discussion

Thus far, the clinical application of conventional targeted therapeutics has been constrained by the reactivation of the targeted signaling pathway or the adoption of alternative signaling pathways [27]. A growing body of evidence suggests that the targeting of transcription factors holds promise as a therapeutic strategy for cancer. In this study, we have identified EGR1 as a transcription factor target in HCC. Our findings indicate that EGR1 expression is downregulated in HCC and that EGR1 inhibits the growth of HCC both in vitro and in vivo. Regarding the examination of EGR1’s antitumor efficacy, our study revealed that EGR1 transcriptionally repressed the expression of PFKL and inhibited PFKL-mediated glycolysis. These findings provide evidence that EGR1 functions as a tumor suppressor in HCC, suggesting that targeting the transcription factor EGR1 holds promise as a viable strategy for HCC treatment.

Increasing evidence indicates that cancer is primarily a metabolic disease characterized by disruptions in the energy metabolism of cancerous cells [28]. The Warburg effect, also known as aerobic glycolysis, plays a pivotal role as the principal energy supplier for cancer cells [29] and is widely acknowledged as a distinctive feature of cancer advancement [30]. EGR1, a transcription factor possessing distinct transcriptional activation and inhibitory domains, demonstrates the capacity to selectively attach to promoter regions of specific genes, thereby regulating gene transcription through either activation or repression [31]. Previous studies have demonstrated that EGR1 assumes intricate and frequently conflicting functions in human malignancies [32–34]. In HCC, EGR1 has been found to have both promoting and inhibitory effects through the activation or repression of various downstream targets [18–20, 35–38]. However, the specific role of EGR1 in cancer metabolism remains uncertain, and previous investigations lacked the genes expression profile detection for mechanistic analysis after EGR1 gene perturbations. In this study, we examined the gene expression profile following alterations in EGR1 expression, including both EGR1 overexpression and knockout. Our analysis using Gene Set Enrichment Analysis (GSEA) revealed a significant downregulation of the glycolysis pathway after EGR1 overexpression, while EGR1 knockout resulted in an upregulation of this pathway. Subsequent experiments provided evidence supporting the inhibitory role of EGR1 in aerobic glycolysis.

Actually, we found that EGR1 exhibited an interaction with the promoter region of PFKL, resulting in the transcriptional downregulation of PFKL expression and subsequent inhibition of aerobic glycolysis. It is worth noting that aerobic glycolysis serves as a primary contributor to cancer development. PFK-1, particularly PFKL, exerts a significant influence on glycolysis during cancer progression. The augmentation of glycolysis, facilitated by PFKL, assumes a crucial role in the malignant advancement of cancer. A study investigating the underlying mechanism of the Warburg effect has revealed that TAp73, a structural homolog of the P53 tumor suppressor, plays a role in stimulating PFKL expression to promote the Warburg effect and enhance cell proliferation [39]. Furthermore, several studies have demonstrated that targeting PFKL to suppress glycolysis exhibits a potent antitumor effect. Recently, utilizing the Drug affinity response target stability (DARTS) method, researchers have identified PFKL as a direct target of penfluridol, which effectively inhibits glycolysis and suppresses esophageal cancer tumorigenesis [40]. Similarly, the study conducted by Yilu Feng et al. demonstrated that E3 ubiquitin ligase A20 interacts with PFKL and facilitates the degradation of PFKL protein, thereby inhibiting glycolysis and proliferation in HCC cell lines [41]. In our study, we identified EGR1 as the transcriptional suppressor of PFKL in HCC. We observed that EGR1 interacted with the promoter region of the PFKL gene, leading to the transcriptional downregulation of PFKL and the subsequent inhibition of glycolysis and proliferation in HCC cells. Our findings contribute to the understanding of the underlying mechanism of aerobic glycolysis mediated by PFKL in HCC. Previous researches have demonstrated that elevated levels of PFKL protein promote the development of HCC [42], while inhibiting PFKL suppresses HCC progression [41, 43]. Our findings align with the previous studies.

Sorafenib serves as the initial systematic therapeutic intervention for advanced HCC patients; however, its therapeutic effectiveness is limited due to the emergence of drug resistance. It has been observed that patients undergoing sorafenib treatment frequently develop resistance to the drug within a six-month timeframe [44]. Recent studies have indicated that glycolysis plays a role in facilitating the development of sorafenib resistance in HCC [45, 46], and some studies have shown that targeting glycolysis can enhance the sensitivity of sorafenib. It has been discovered that inhibiting pyruvate kinase M2 (PKM2), which plays a crucial role in the glycolytic pathway by catalyzing the final step of glycolysis, can restore sensitivity of HCC cells to sorafenib therapy [47, 48]. Furthermore, Wang et al. demonstrated that the combination of the glycolysis inhibitor 2-DG and sorafenib resulted in a more effective therapeutic outcome for HCC [49]. In our study, it was observed that the downregulation of EGR1 resulted in an increase in the expression of PFKL, thereby enhancing glycolysis and subsequently leading to resistance to sorafenib. Furthermore, the overexpression of EGR1 enhanced the sensitivity of HCC cells to sorafenib. Conversely, the excessive expression of PFKL promoted the progression of sorafenib resistance in HCC cells (Fig. S6A). Additionally, the combination of EGR1 and sorafenib further suppressed the proliferation of HCC cells and the growth of HCC tumors. Our research suggests that the combination of EGR1 and sorafenib could potentially serve as a promising therapeutic approach for individuals compared to single sorafenib treatment.

Notably, our study revealed a decrease in EGR1 mRNA and protein expression in both HCC clinical samples and HCC cells. More importantly, the expression of EGR1 in HCC cells was further downregulated following treatment with sorafenib. According to previous reports, EGR1 can be activated in response to various cellular stimuli, such as ionizing radiation, growth factors, reactive oxygen species, inflammatory factors, tumor necrosis factor, and other factors [50–55]. The transcription of EGR1 is contingent upon the signal transduction pathway involving RAS, RAF, MEK1/2, and ERK1/2 [56]. However, previous investigations lack resolution regarding the underlying factors contributing to the decline in EGR1 expression in HCC. Our research results demonstrate a significant reduction in EGR1 expression in HCC, which subsequently led to the discovery of its suppressive effect on HCC growth upon further analysis. Regrettably, our findings did not yield a definitive answer to the reasons for EGR1 downregulation in HCC. Consequently, there is a strong anticipation for future research endeavors to elucidate this matter and bolster the development of novel therapeutic strategies for HCC.

## Conclusion

In conclusion, our findings indicate that EGR1 exerts an inhibitory effect on HCC growth both in vitro and in vivo. This inhibition is achieved through the binding of EGR1 to the promoter region of PFKL, leading to the transcriptional suppression of PFKL and subsequent inhibition of the glycolysis process. Additionally, the downregulation of EGR1 is associated with resistance to sorafenib, while the overexpression of EGR1 or EGR1 gene therapy enhances the sensitivity of HCC cells and xenografted tumors to sorafenib. These results suggest that EGR1 possesses an anti-tumor function in HCC, highlighting the potential of EGR1 gene therapy as a therapeutic approach.

### Supplementary Information

**Additional file 1.**

## Data Availability

The data from the RNA sequencing analysis used in this research can be found in the GEO database (GSE238116).

## Abbreviations

EGR1: Early growth response 1
HCC: Hepatocellular Carcinoma
PFKL: Phosphofructokinase, liver type
KO: Knockout
ECAR: Extracellular acidification rate
DEGs: Differential expression genes
TCGA: The Cancer Genome Atlas
GSEA: Gene Set Enrichment Analysis
ALT: Alanine aminotransferase
AST: Aspartate aminotransferase
GDSC: Genomics of drug sensitivity in cancer
