# Supplementary material for: EGR1 suppresses HCC growth and aerobic glycolysis by transcriptionally downregulating PFKL
Source: J Exp Clin Cancer Res. 2024 Jan 29;43:35. doi: 10.1186/s13046-024-02957-5 (PMC10823730; doi:10.1186/s13046-024-02957-5)
Supplement: Supplementary file 1 — Additional file 1. [file 13046_2024_2957_MOESM1_ESM.docx]

**EGR1 suppresses HCC growth and aerobic glycolysis by transcriptionally downregulating PFKL**

Mingang Pan^1, †^, Muyu Luo^1, †^, Lele Liu^1, †^, Yunmeng Chen^1^, Ziyi Cheng^1^, Kai Wang^1^, Luyi Huang^1^, Ni Tang^1^, Jianguo Qiu^2*^, Ailong Huang^1*^, Jie Xia^1*^

**Table S1**

| **Antibodies** | | | | | |
| --- | --- | --- | --- | --- | --- |
| **Catalog** | **Antibody**  **Name** | **Antibody**  **species** | **MW（KD）** | **Company** | **Application and Radio** |
| ab194357 | EGR1 | R | 75 | Abcam | WB 1:10000  IHC 1:100 |
| ab307199 | EGR1 | R | 75 | Abcam | CHIP |
| ab181064 | PFKL | R | 75 | Abcam | WB 1:1000 |
| gb111136 | PFKL | R | 75 | Servicebio | IHC1:1000 |
| TA-08 | GAPDH | M | 37 | ZSGB-BIO | WB 1:2000 |
| PA5-16785 | Ki67 | M |  | Invitrogen | IHC 1:200 |
| TA-09 | ACTB | M | 45 | ZSGB-BIO | WB 1:2000 |
| **Compounds** | | | | | |
| **Compounds name** | | **Catalog** | | **Company** | |
| sorafenib | | S7397 | | Selleck | |
| 2-DG | | [HY-13966](https://www.medchemexpress.cn/2-Deoxy-D-glucose.html) | | MCE | |
| Oligomycin | | MZ8001 | | MKBio | |
| **Primers’ sequence** | | | | | |
| EGR1-forward | | | CAGCAGCAGCACCTTCAAC | | |
| EGR1-reverse | | | GTCTCCACCAGCACCTTCTC | | |
| PFKL-forward | | | CATGCTGTGGTTGTCGGAGAA | | |
| PFKL-reverse | | | GCCACCGCCTTCTTCTTCAG | | |
| ACTB-forward | | | TCGTGCGTGACATTAAGGAGAA | | |
| ACTB-reverse | | | AGGAAGGAAGGCTGGAAGAGT | | |
| CHIP-P1-forward | | | GGATGCCTCAGGGGTAGACACAG | | |
| CHIP-P1-reverse | | | ACGGTGGAAATGGTGGCTGTG | | |
| CHIP-P2-forward | | | GGTCCCCGGCCACATCCTC | | |
| CHIP-P2-reverse | | | ACCTTCCCGCTGTGCGTTTC | | |


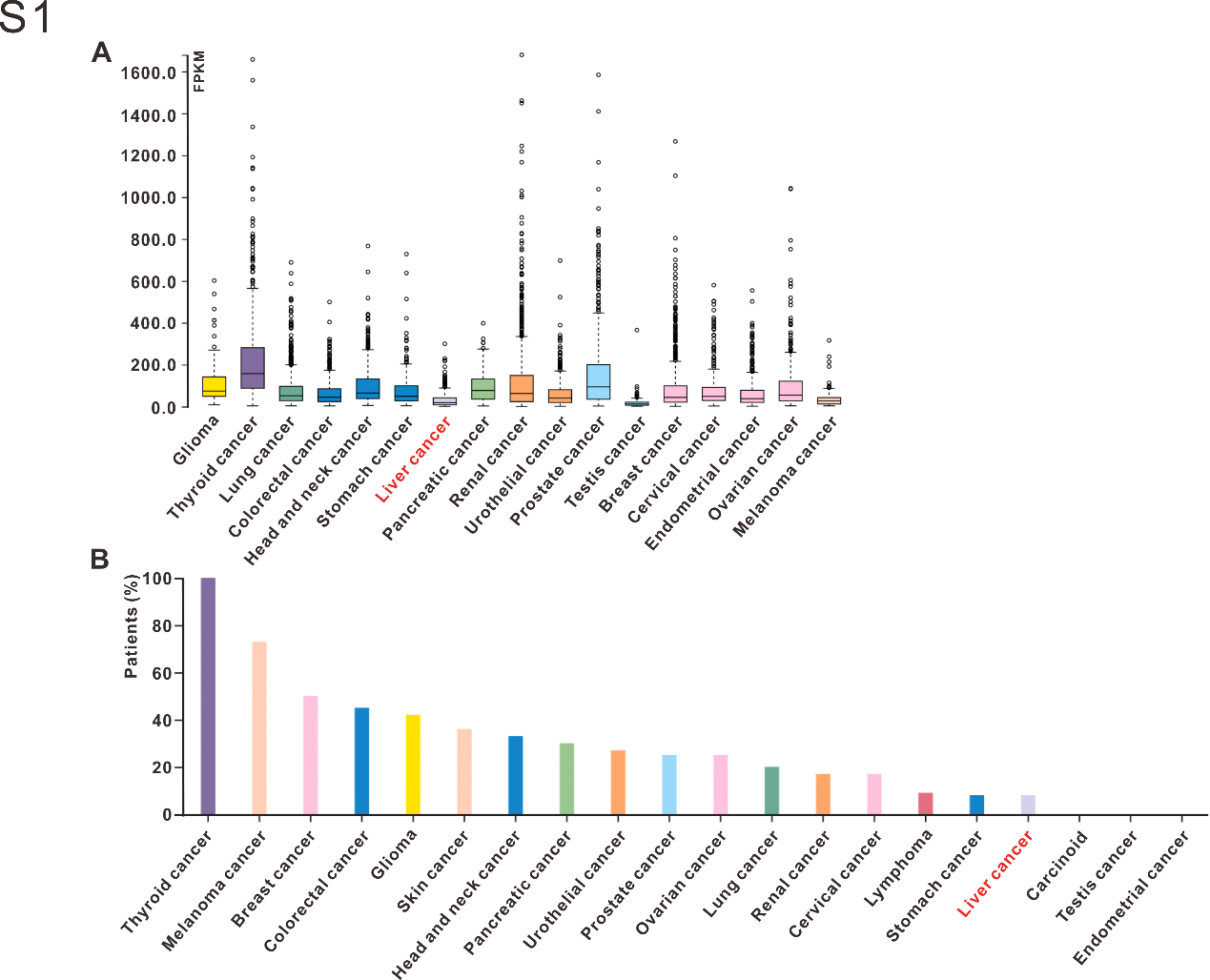


**Figure S1** The pan-cancer analysis showed the low levels of EGR1 in HCC among various cancer types. **(A)**Pan-cancer analysis of EGR1 mRNA expression in multiple cancer types based on TCGA database. **(B)**Pan-cancer analysis of EGR1 protein expression in multiple cancer types based on the human protein atlas database.


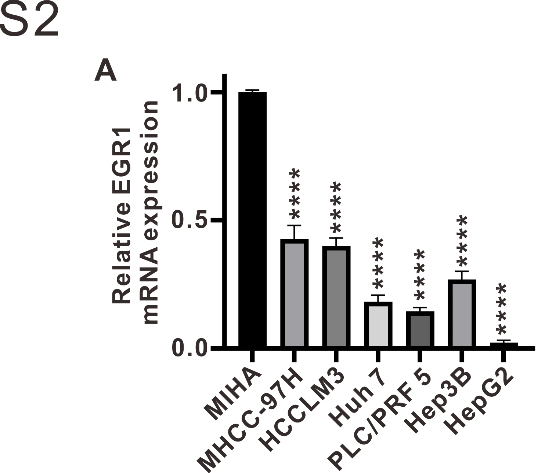


**Figure S2** EGR1 mRNA expression was downregulated in HCC cells compared to normal liver cell line MIHA. (A) The mRNA expression of EGR1 in HCC cell lines and normal liver cell line MIHA was detected by RT-qPCR. ****P < 0.0001.


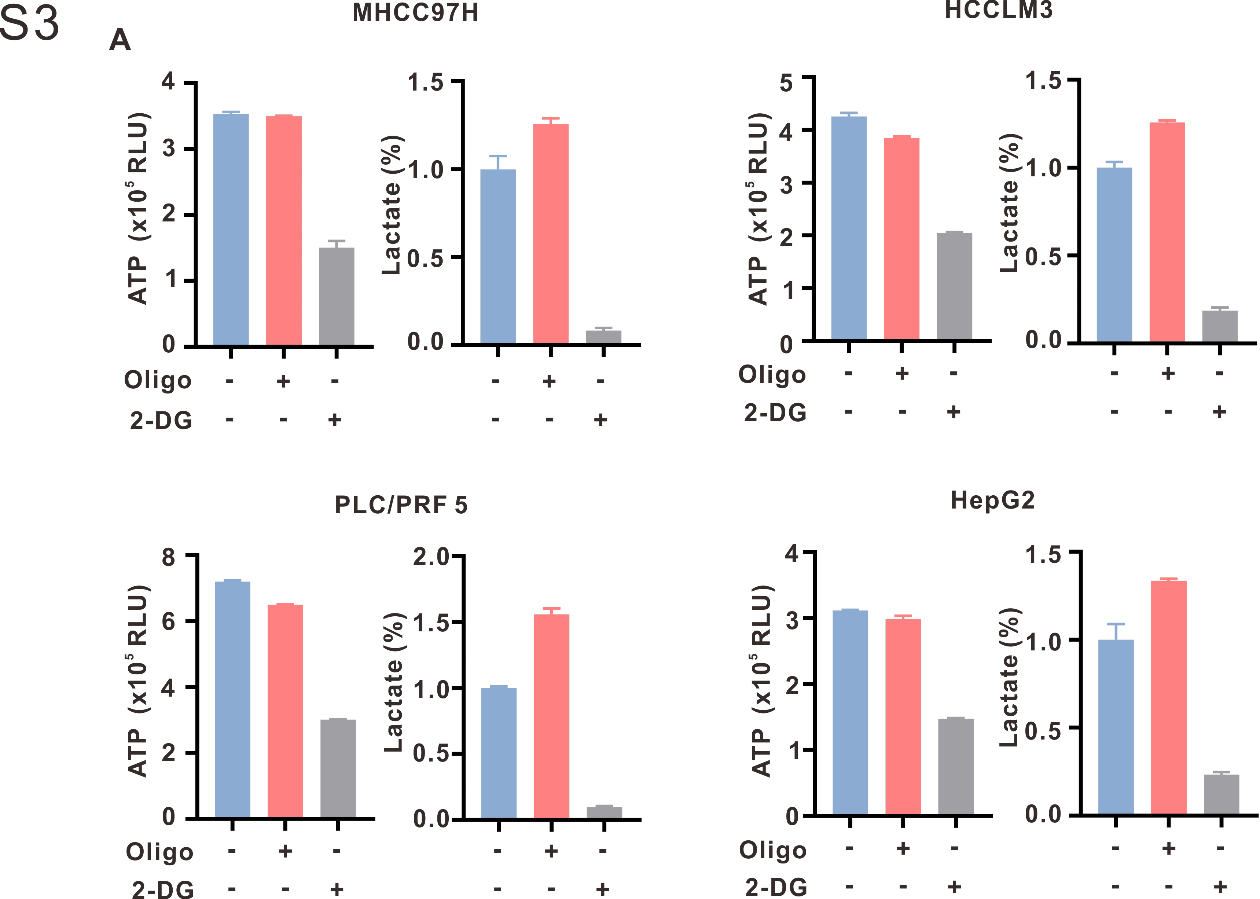


**Figure S3** HCC cells are highly dependent on glycolysis for generation of ATP in our experimental conditions. (A) The analysis of metabolic pathway dependency was evaluated based on ATP and lactate levels. The alterations observed in lactate levels indicate that the suppression of oxidative phosphorylation triggers the activation of the glycolytic pathway. The administration of glycolysis inhibitors leads to a reduction in ATP levels, whereas the inhibition of oxidative phosphorylation does not significantly impact ATP levels. These findings suggest a strong reliance of the cellular metabolism on the glycolytic pathway. Oligomycin, oligo.


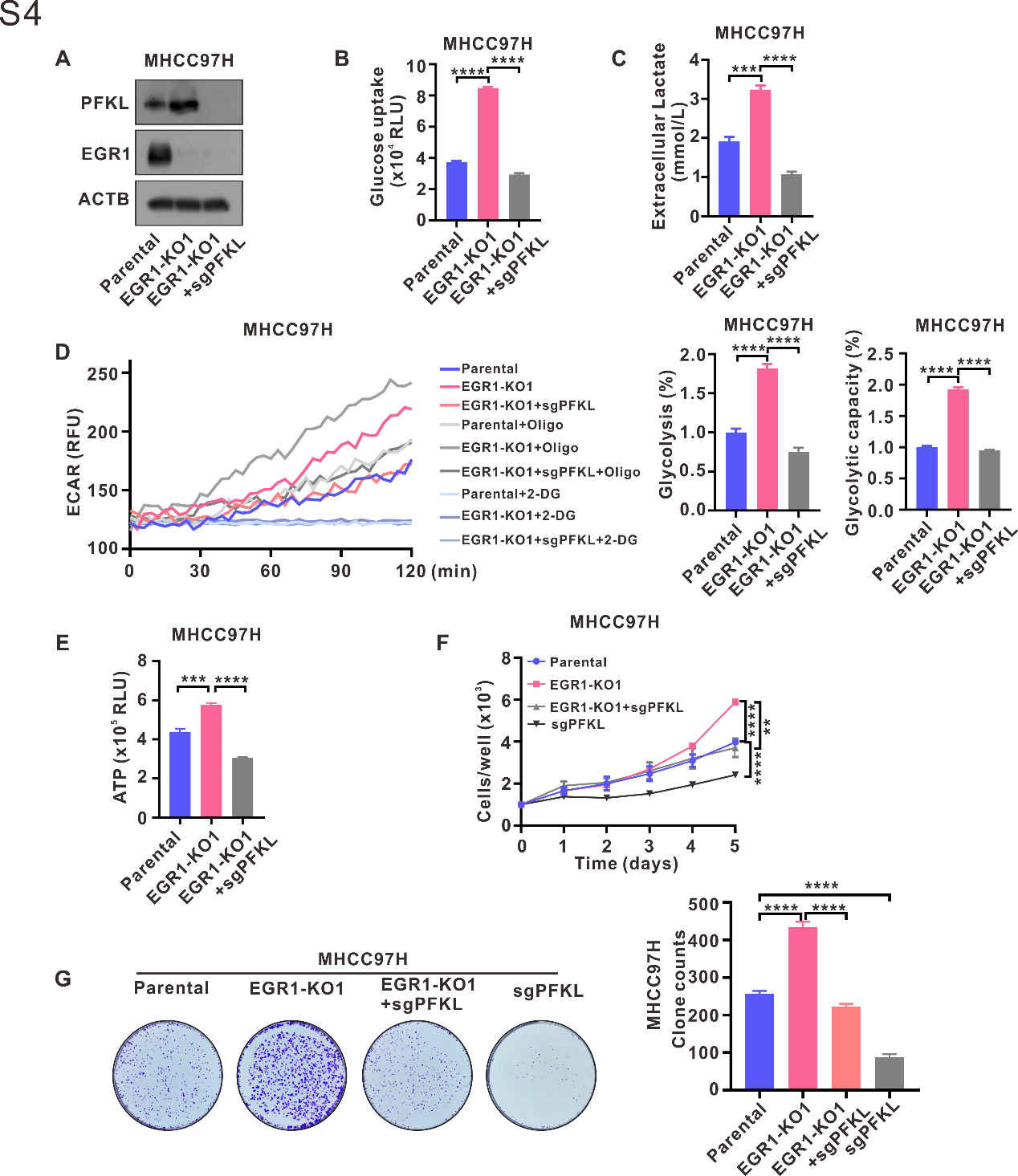


**Figure S4** EGR1 knockout promoted HCC cells proliferation by upregulating PFKL-mediated aerobic glycolysis. (A)The WB analysis was conducted on PFKL and EGR1 in EGR1 knockout MHCC97H cells following PFKL silence. (B) (C) (D) (E) (F) (G) PFKL knock down was found to counteract the enhanced effects of EGR1 knockout on glucose uptake, extracellular lactate levels, glycolysis and glycolytic capacity, ATP levels, cell proliferation and colony formation capacity. **P < 0.01, ***P < 0.001, ****P < 0.0001.


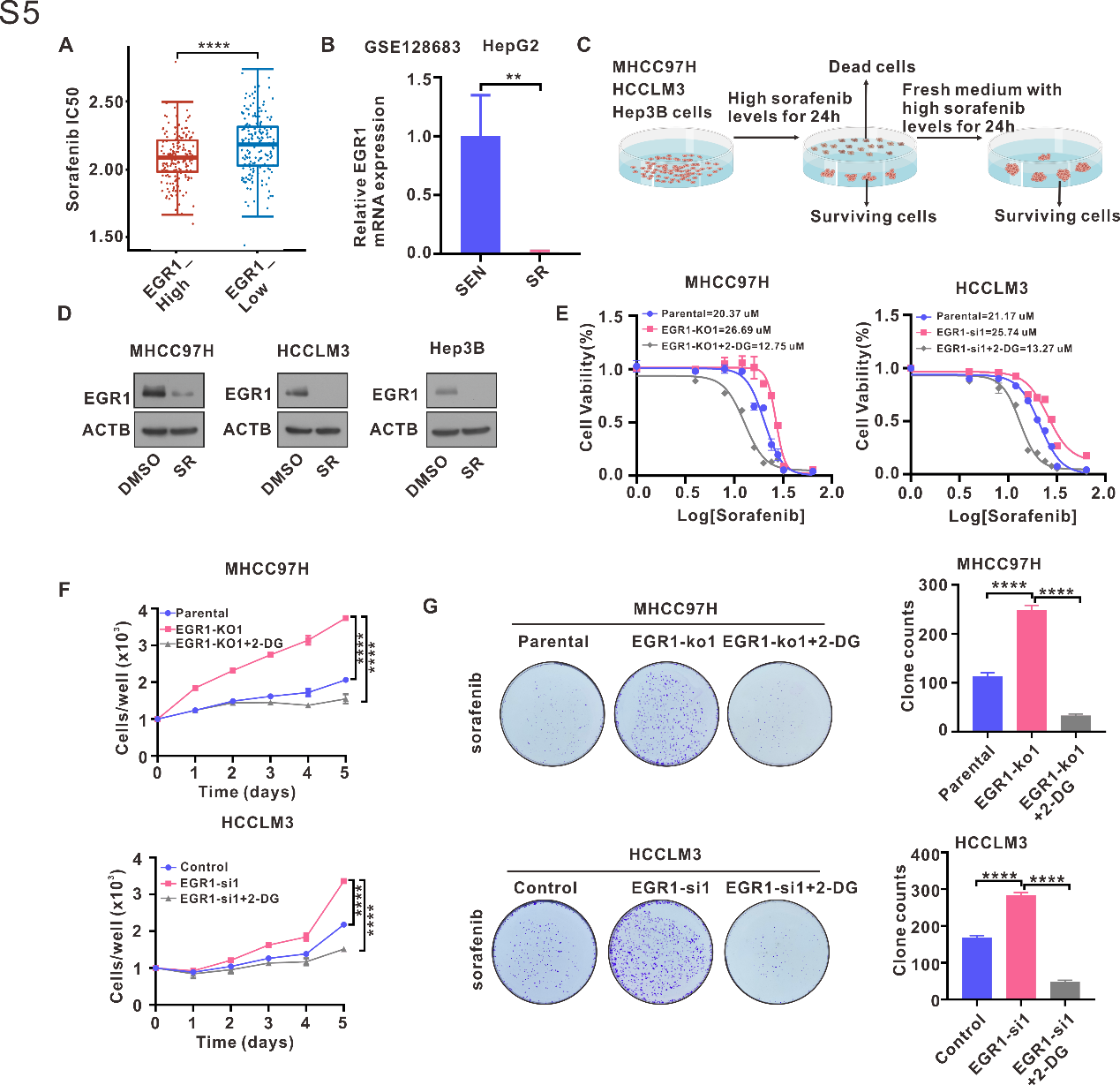


**Figure S5** EGR1 downregulation promoted sorafenib resistance by upregulating aerobic glycolysis in HCC cells. (A)The IC50 of sorafenib was predicted in GSDC database based on EGR1 expression. (B)The expression of EGR1 mRNA in sorafenib-resistant HepG2 cells was analyzed using the GSE128683 dataset. (C)The schematic representation that HCC cells were treated with sorafenib. (D)Western blot analysis was conducted to assess the expression of EGR1 in HCC cells that survived after treatment with sorafenib. (E)The IC50 values of sorafenib were determined in EGR1 knockout MHCC97H and EGR1 silencing HCCLM3 with or without 2-DG treatment and the results showed EGR1 downregulation led to an elevation in IC50 values of sorafenib in MHCC97H and HCCLM3 cells, whereas 2-DG abrogated the increased IC50 values of sorafenib in EGR1 knockout MHCC97H cells and EGR1 silencing HCCLM3 cells. (F) (G) In the context of sorafenib, IncuCyte zoom cell proliferation assays and colony formation assays were performed in EGR1 knockout MHCC97H and EGR1 silencing HCCLM3 with or without 2-DG treatment and the results showed EGR1 downregulation promoted cell proliferation and colony formation capacity in MHCC97H and HCCLM3 cells, whereas 2-DG abrogated the increased cell proliferation and colony formation capacity in EGR1 knockout MHCC97H cells and EGR1 silencing HCCLM3 cells. **P < 0.01, ****P < 0.0001.


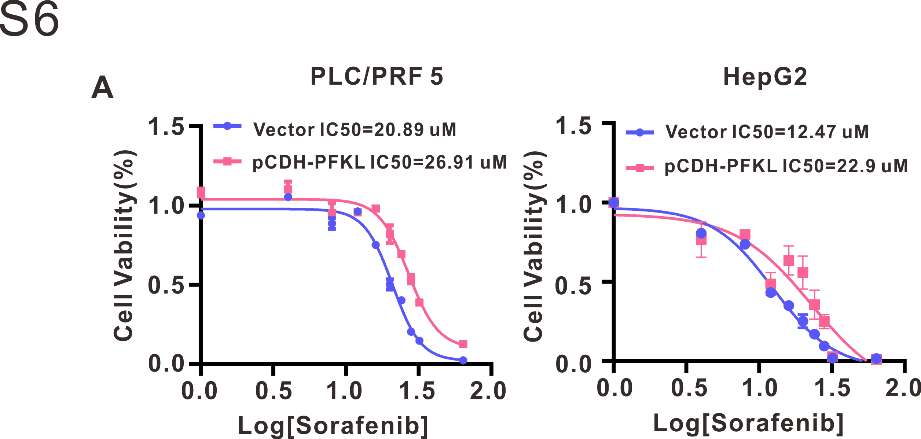


**Figure S6** PFKL overexpression promoted sorafenib resistance. (A)The IC50 values of sorafenib was determined in PLC/PRF5 and HepG2 cells after PFKL overexpression.
